# Transarterial chemoembolization plus sorafenib versus sorafenib for intermediate–advanced hepatocellular carcinoma: A meta-analysis comparing clinical outcomes

**DOI:** 10.1097/MD.0000000000026958

**Published:** 2021-08-20

**Authors:** Yong Xie, Huan Tian, Bin Xiang, Yongjin Zhang, Jian Liu, Zhuoyan Cai, Hua Xiang

**Affiliations:** a Department of Interventional Radiology and Vascular Surgery, the First Affiliated Hospital of Hunan Normal University, Changsha, P. R. China; b Department of Radiology, the Second Affiliated Hospital of Hebei Medical University, Shijiazhuang, P. R. China.

**Keywords:** hepatocellular carcinoma, intermediate-advanced, meta-analysis, sorafenib, transarterial chemoembolization

## Abstract

**Background:**

Hepatocellular carcinoma (HCC) ranks as the sixth most common cancer and the second leading cause of cancer-related death worldwide, local and systemic therapies are beneficial for those who have more advanced disease or are not suitable for radical treatment. We aim to investigate the clinical outcomes of transarterial chemoembolization (TACE) plus sorafenib compared with sorafenib monotherapy for intermediate–advanced HCC.

**Methods:**

A systematic search according to preferred reporting items for systematic reviews and meta-analyses guidelines in the PubMed database was conducted from inception to December 31, 2020 for published studies comparing survival outcomes and tumor response between TACE + sorafenib and sorafenib alone for intermediate–advanced HCC.

**Results:**

Five eligible cohort studies and a randomized controlled trial with a total of 3015 patients were identified. We found that the TACE + sorafenib group had a significantly better overall survival (OS) (hazard ratio, 0.77; 95% confidence interval [CI] 0.66–0.88, *P* < .001) than those treated with sorafenib. Median OS ranged from 7.0 to 22.0 months with TACE + sorafenib and from 5.9 to 18.0 months with sorafenib. The combination of TACE + sorafenib had a significantly better time to progression (hazard ratio, 0.74; 95% CI 0.65–0.82, *P* < .001) than those treated with sorafenib. Median time to progression ranged from 2.5 to 5.3 months with TACE + sorafenib and from 2.1 to 2.8 months with sorafenib. The results showed the TACE + sorafenib group had a higher disease control rate (log odds ratio, 0.52; 95% CI 0.25–0.80, *P* = .0002), objective response rate (log odds ratio, 0.85; 95% CI 0.37–1.33, *P* = .0006) than sorafenib group. Hand–foot skin reaction, diarrhea, fatigue, vomiting, and alanine aminotransferase (ALT) elevation were common adverse events. The adverse events were similar between the 2 groups excluding elevated ALT.

**Conclusion:**

Although the TACE + sorafenib group had a higher elevated ALT, the combination of TACE + sorafenib had an OS benefit compared with sorafenib in the treatment of intermediate–advanced HCC. Further research is necessary to affirm this finding and clarify whether certain subgroups benefit from different combinations between TACE and sorafenib.

## 1 Introduction

Primary liver cancer (PLC) is a common malignant tumor of the digestive system worldwide. According to the data released by GLOBOCAN 2018, the number of new cases of liver cancer worldwide is as high as 841,000, ranking sixth in malignant tumors, and the death toll is as high as 782,000, ranking second in malignant tumors.^[1,2]^

The main pathological type of PLC is hepatocellular carcinoma (HCC), accounting for 80% to 90%; while the other types include intrahepatic cholangiocarcinoma (10%–20%) and HCC–intrahepatic cholangiocarcinoma mixed type.^[3–5]^ They differ greatly in pathogenesis, biological behavior, molecular characteristics, clinical manifestations, histopathological morphology, diagnosis, treatment methods, and prognosis. The incidence of HCC is highest in Eastern Asia and sub-Saharan Africa, followed by countries in Southern and Western Europe, North and Central America, and the lowest incidence is observed in Northern Europe and South Central Asia.^[6,7]^

The occurrence of HCC is a complex process caused by many risk factors. The dominant factors include chronic hepatitis viruses (mainly caused by hepatitis B virus and/or hepatitis C virus infection), and chronic hepatitis (alcoholic liver disease), diabetes mellitus, and nonalcoholic fatty liver,^[8]^ eating aflatoxin-contaminated food, schistosomiasis. Fortunately, there are multiple treatment options for HCC. The treatment strategies for HCC recommended in the clinical guidelines include surgical resection, ablation, liver transplantation, transarterial chemoembolization (TACE), radiotherapy, and systemic therapies. Surgical resection, TACE are primarily used in localized HCC, while systemic therapies are used in metastatic disease and radiotherapy can be used in localized but also used palliatively in advanced disease. In recent years, new treatment strategies, such as immunotherapy and molecular targeting therapy, have been widely used in the treatment of HCC worldwide.^[9–11]^ These means are mainly aimed at improving the prognosis of patients by reducing the local tumor burden and related symptoms. Although radical treatment is the preferred treatment method because it provides a chance of cure and better long-term results, only a few patients have the opportunity to receive this treatment.^[12]^

According to the Barcelona clinical liver cancer staging system, which has been widely recognized by international peers and has been commonly used in clinical practice,^[3,13,14]^ TACE is recommended as the first-line treatment for intermediate-stage HCC (Eastern Cooperative Oncology Group performance status 0, multinodular, Child–Pugh A/B). Specific methods include conventional TACE, and drug-eluting beads for TACE. A lot of researchers show that TACE is a well-established treatment for this stage of HCC.^[9,15]^

Sorafenib blocks the expression of VEGFR, RET, PDGFR, and c-KIT, and inhibits the downstream Raf serine/threonine kinase activity to prevent tumor growth and recurrence. The European Association for the Study of the Liver guidelines recommends sorafenib as the first-line treatment for advanced liver cancer.^[3,9]^

So far, a few studies have compared the clinical outcomes of TACE + sorafenib and sorafenib, and these studies have yielded conflicting results. Some studies found no significant difference in survival benefits, while one of the larger studies found survival benefits related to TACE + sorafenib.^[16–21]^

Our study aimed to compare the clinical outcomes and survival benefits between TACE + sorafenib and sorafenib alone for intermediate–advanced HCC. To our knowledge, this is the first meta-analysis on this topic and represents the largest patient population (involving 3015) analyzed so far.

## 2 Methods

### 2.1 Study protocol

This meta-analysis was carried out by using the protocol designated by the Cochrane collaboration^[22]^ and reported based on the items of preferred reporting items for systematic reviews and meta-analyses.^[23]^ The review protocol has been registered on the PROSPERO (https://www.crd.york.ac.uk/PROSPERO/). Published articles were chosen; hence, an ethical review was not required.

### 2.2 Search strategy

Two investigators (Y.X and H.T) independently searched the literature published in PubMed from inception to December 31, 2020. The main search terms used for the search were “hepatocellular carcinoma,” “HCC,” “transcatheter arterial chemoembolization,” “transarterial chemoembolization,” “TACE,” and “sorafenib.” There was no language restriction for this search, and the reference list of all selected articles will be filtered to identify other studies.

### 2.3 Selection criteria

The inclusion criteria:

(1) Patients: patients with intermediate–advanced HCC (confirmed by pathologically or cytologically or diagnosed by computed tomography or magnetic resonance imaging).
(2) Iintervention and comparison: TACE + sorafenib versus sorafenib monotherapy.
(3) Outcomes: overall survival (OS), time to progression (TTP), tumor response rate, adverse events.
(4) Sstudy: randomized controlled trials (RCT) or cohort studies.

The exclusion criteria:

(1) Articles with missing data or articles containing only abstract or duplicate data.
(2) Letters, meta-analyses, reviews, comments, animal trials, or meeting articles.
(3) Single-arm articles.

### 2.4 Data extraction

We extracted data using standardized forms and extracted the following data: first author's name, publication year and publication journal, study design, country, baseline characteristics of the patient population and Barcelona clinical liver cancer stage, median follow-up, etiology of HCC, tumor response evaluation criteria, and adverse event evaluation criteria; treatment procedure; and the primary outcomes of this study were OS and TTP. The secondary outcomes were tumor response and adverse events. In some of the included trials, the tumor response was recorded according to the modified guidelines for the response evaluation criteria in solid tumours^[24]^ for HCC. In the base of the degree of tumor regression, the efficacy could be evaluated as complete response; partial response; stable disease; progressive disease; disease control rate (DCR); and objective response rate (ORR).

### 2.5 Quality assessment

We assessed the quality of each included RCT according to the Cochrane risk of bias tool,^[25]^ which involves the following 6 parts: random allocation; allocation concealment; blinding method; completeness of results; selective reporting of research results; and other sources of bias. According to the following criteria, the included trials are classified as low quality, high quality, or moderate quality^[26]^:

a) as long as either random allocation or allocation concealment is a high risk, the trial is considered low quality;
b) if random allocation and allocation concealment are all assessed as low risk of bias, and other items are a nonhigh-risk, the trial is considered high quality;
c) if the trial does not meet any of the above a and b, then, it is considered to be of moderate quality.

The Newcastle–Ottawa Scale,^[27]^ which is consists of 3 main items (ie, comparability, exposure, and selection), was adopted to assess the quality of the cohort studies. Articles with a score of 6 to 9 were considered high quality.^[27]^

### 2.6 Statistical analysis

For TTP and OS, the hazard ratio (HR) with 95% confidence interval (CI) were combined while the effect sizes of tumor response and adverse events were pooled by log odds ratio (OR) with 95% CI. Statistical heterogeneity between studies was examined using Cochran *Q* test, while Higgin *I*^2^ statistic was calculated to quantify the heterogeneity of the included trials. In the absence of statistical heterogeneity (*P* > .10 and *I*^2^ < 50%), we used a fixed-effects model to pool the results. Otherwise, we presented the results employing the random-effects model. If necessary, we performed a sensitivity analysis to explore the sources of heterogeneity and the results’ stability. All *P* values (2-sided) with *P* < .05 are considered to be statistically different. We used statistical software Stata16 (Stata Corporation, College Station, TX) for all statistical analyses.

## 3 Results

### 3.1 Study selection and quality assessment

**Figure 1:**
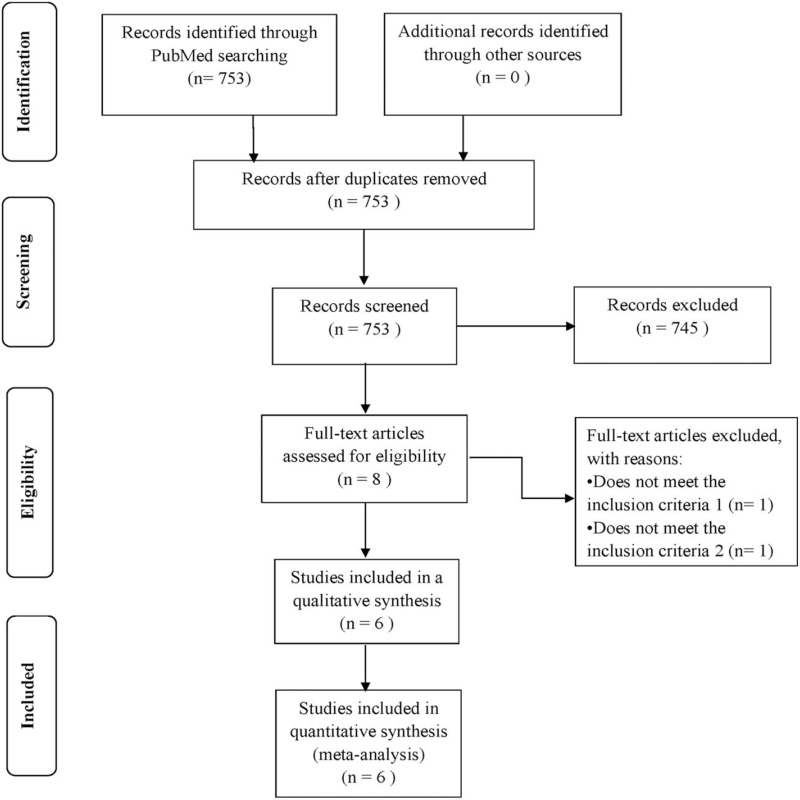
PRISMA flow diagram of the study selection process. PRISMA = preferred reporting items for systematic reviews and meta-analyses.

The study identified 753 published pieces of literature in our initial extensive search. After carefully reading titles and abstracts, 8 articles were selected for possible inclusion. Then, after a review of full text, 5 eligible cohort studies, and an RCT published between 2013 and 2020 were found to meet the inclusion criteria (Fig. 1). The meta-analysis included 3015 patients with intermediate–advanced HCC. Among the 3015 patients, 861 received TACE + sorafenib treatment, and 2154 received sorafenib treatment. The proportion of males was 68.8% to 94.4% and the sample size ranged from 8 to 1686 patients. The number of patients with Eastern Cooperative Oncology Group 0 ranges from 0 to 140; Child–Pugh class A, 34 to 148; median AFP, ranged from 65 ng/mL to 24,113.9 ng/mL (as summarized in Table 1). We included an RCT in total and evaluated it according to the Cochrane Collaboration's bias risk evaluation criteria.^[25]^ In the item of random allocation, it is difficult to judge whether it is correct or not because the study did not mention the specific details. Herein, it is judged as uncertain; in the item of allocation concealment, the researcher adopts an open-label, so it is judged as high risk; in the item of the blind method, the researcher uses the blind method for the result measurer, and does not use blind method for the main researcher, which may lead to bias; in the item of result integrity, the research results are complete; in the item of selective reporting of research results, there is a clear research plan, and the systematic review of the designated outcomes (primary and secondary results) are reported; in the item of other sources of bias, the information is incomplete, and it is difficult to judge whether there is an important bias. Moreover, we applied Newcastle–Ottawa Scale^[27]^ to evaluate 5 cohort studies, 3 studies received 9 stars, 1 study received 8 stars, and 1 study received 5 stars. In summary, the quality, therefore, of the studies included in this analysis was assessed to be good (n = 4) or fair (n = 2). The specific details of the research quality assessment are listed in Table 2.

**Table 1:** Characteristics of included studies.

First author, year	Publication	Study design/country	Periods	Intervention	Patients (n)	Male (n)	Age (yr)	BCLC stage (n)	Vascular invasion status (n)		AFP (ng/mL)	Median follow-up (mo/d)	Tumor response evaluation criteria	Adverse event evaluation criteria
									No	Yes				
Joong-Won Park/2019^[19]^	Journal of Hepatology	RCT/South Korea	January 2013 and December 2015	S + T	170	136	60.2 (9.6) mean (SD)	A:3 B:39 C:128	102	68	7557.1 (22,642.52) mean (SD)	14 (90% CI, 9.4–20.2) [IQR], 4.0 to 27.1	RECIST version 1.1 criteria	National Cancer Institute Common Terminology Criteria for Adverse Events version 3.0.
				S	169	147	61.3 (9.6) mean (SD)	A:0 B:44 C:125	106	63	24,113.9 (168,194.81) mean (SD)	18.7 (90% CI, 11.1–23.3) (IQR, 2.3 to 27.1)		
Yingqiang Zhang/2015^[17]^	The Oncologist	Retrospective cohort/China	January 2009 and June 2013	S + T	45	43	50.1 (8.8) mean (SD)	NR	NR	NR	NR	7.3 (range: 2–18)	Modified RECIST	National Cancer Institute Common Terminology Criteria for Adverse Events version 3.0.
				S	44	41	53.6 (9.7) mean (SD)	NR	NR	NR	NR			
Gwang Hyeon Choi/2013^[16]^	Radiology	Retrospective cohort/Korea	April 2007 and July 2011	S + T	164	139	52 (26–75) median, range	NR	106	58	NR	6.9 (range, 1.4–34.6)	Modified RECIST	National Cancer Institute Common Terminology Criteria for Adverse Events version 4.0
				S	191	166	54 (22–84) median, range	NR	94	97	NR	4.9 (range, 1.4–43.6)		
Victor C. Kok/2019^[20]^	Cancers	Retrospective cohort/nationwide	August 2012, and 31 December 2013	S + T	426	355	60.4 (50.7–68.7) median, IQR	NR	NR	NR	NR	221 d (quartile, 140–345)	Modified RECIST	NR
				S	1686	1410	60.0 (51.8–67.8) median, IQR	NR	NR	NR	NR	133 d (quartile, 68–251)		
Yusuke Kimura/2020^[21]^	Asian Pacific Journal of Cancer Prevention	Retrospective cohort/Japan	April 2009 and June 2018	S + T	8	5	75.3 (69.9–75.3) median, IQR	A:0 B:5 C:3	NR	NR	65 (8.8–7613) median (IQR)	NR	Modified RECIST	National Cancer Institute Common Terminology Criteria for Adverse Events version 5.0
				S	8	6	73.5 (65.8–78.1) median, IQR	A:0 B:6 C:2	NR	NR	10.8 (7.2–89,872) median (IQR)	NR		
Fei-Xiang Wu/2017^[18]^	BMC Cancer	Retrospective cohort/China	August 2004 and November 2014	S + T	48	46	47.6 (12.73) mean (SD)	A:0 B:16 C:32	22	26	172.5 (1.4, 4000) median (range)	NR	Modified RECIST	National Cancer Institute Common Terminology Criteria for Adverse Events version 3.0.
				S	56	48	50.23 (11.88) mean (SD)	A:0 B:10 C:46	19	37	400 (0.78, 12,100) median (range)	NR		

AFP = alpha-fetoprotein, BCLC = Barcelona clinical liver cancer, IQR = interquartile range, NR = not reported, RCT = randomized controlled trial, RECIST = response evaluation criteria in solid tumours, S+T = TACE + sorafenib, S = sorafenib, SD = standard deviation.

**Table 2:** Quality assessment of all cohort studies using Newcastle–Ottawa Scale (NOS).

	Selection					Outcome			
First author, year	Representativeness of the exposed cohort	Selection of the nonexposed cohort	Ascertainment of exposure	Demonstration that outcome of interest was not present at start of study	Comparability	Assessment of outcome	Was follow-up long enough for outcomes to occur	Adequacy of follow-up of cohort	Summary
Gwang Hyeon Choi, 2013^[16]^	^∗^	^∗^	^∗^	^∗^	^∗∗^	^∗^	^∗^	^∗^	9
Yingqiang Zhang, 2015^[17]^	^∗^	^∗^	^∗^	^∗^	^∗∗^	^∗^	^∗^	^∗^	9
Fei-Xiang Wu, 2017^[18]^	^∗^	^∗^	^∗^	^∗^	^∗^	^∗^	^∗^	^∗^	8
Victor C. Kok, 2019^[20]^	^∗^	^∗^	^∗^	^∗^	^∗∗^	^∗^	^∗^	^∗^	9
Yusuke Kimura, 2020^[21]^		^∗^	^∗^		^∗^		^∗^	^∗^	5

### 3.2 Overall survival

**Figure 2:**
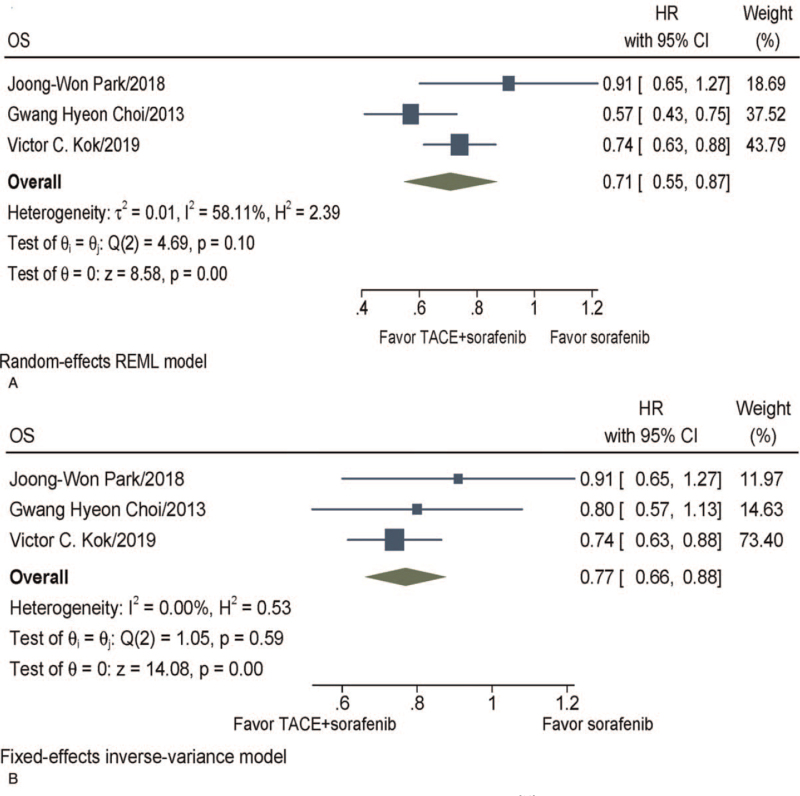
Meta-analysis of OS. (A) Pooled results before the propensity score matching of Choi et al.^[16]^ (B) Pooled results after the propensity score matching of Choi et al.^[16]^ CI = confidence interval, HR = hazard ratio, OS = overall survival, TACE = transarterial chemoembolization.

Three studies^[16,19,20]^ reported OS. Meta-analysis showed that the median OS ranged from 7.0 to 22.0 months with TACE + sorafenib and from 5.9 to 18.0 months with sorafenib. Patients in the TACE + sorafenib combination therapy group had significantly longer OS (HR, 0.71; 95% CI 0.55–0.87, *P* < .001) than those treated with sorafenib. Heterogeneity was found in these studies (*I*^2^ = 58.11%, *P* = 0.1), so a random-effect model was applied to calculate the combined HR (Fig. 2A). Considering that 1 study^[16]^ used propensity score matching, we pooled its survival results with other studies again, and the results indicated that the combination group TACE + sorafenib had significantly longer OS (HR, 0.77; 95% CI 0.66–0.88, *P* < .001) than those treated with sorafenib. As no significant heterogeneity was observed (*I*^2^ = 0%, *P* = .59), a fixed-effect model was applied to estimate the pooled HR (Fig. 2B).

### 3.3 Time to progression

**Figure 3:**
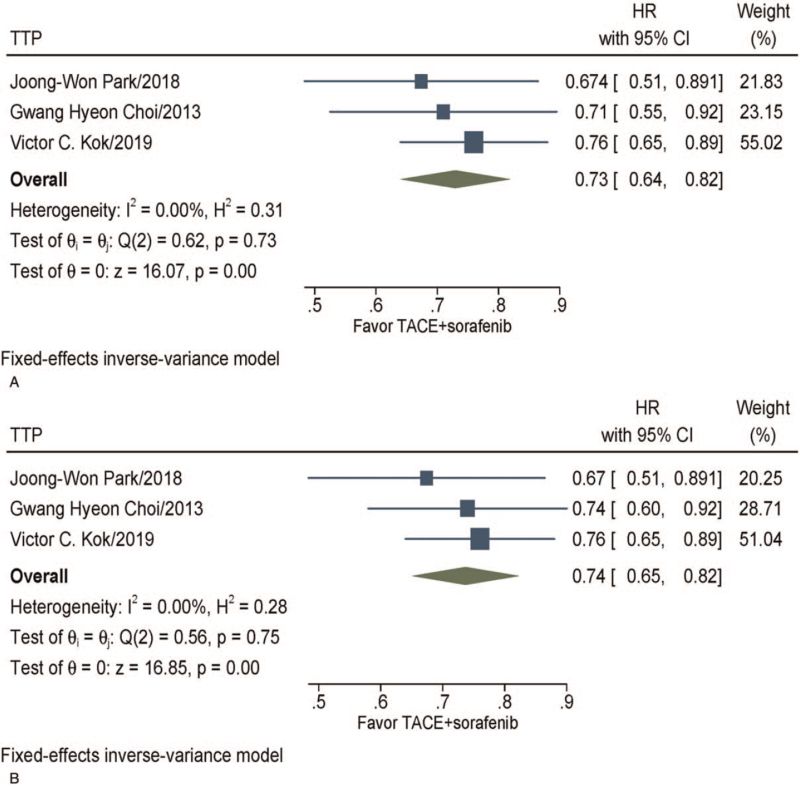
Meta-analysis of TTP. (A) Pooled results before the propensity score matching of Choi et al.^[16]^ (B) Pooled results after the propensity score matching of Choi et al.^[16]^ CI = confidence interval, HR = hazard ratio, TACE = transarterial chemoembolization, TTP = time to progression.

The TTP were reported in 3 trials.^[16,19,20]^ Meta-analysis showed that the median TTP ranged from 2.5 to 5.3 months with TACE + sorafenib and from 2.1 to 2.8 months with sorafenib. The combination group TACE + sorafenib had significantly longer TTP (HR, 0.73; 95% CI 0.64–0.82, *P* < .001) than those treated with sorafenib. As significant homogeneity was observed (*I*^2^ = 0%, *P* = .73), a fixed-effect model was adopted to estimate the combined HR (Fig. 3A). Similarly, considering that 1 study^[16]^ used propensity score matching, we pooled its TTP results with other studies again, and the results indicated that TACE + sorafenib had significantly longer TTP (HR, 0.74; 95% CI 0.65–0.82, *P* < .001) than those treated with sorafenib. As no significant heterogeneity was observed (*I*^2^ = 0%, *P* = .75), a fixed-effect model was employed to estimate the pooled HR (Fig. 3B).

### 3.4 Tumor response

**Figure 4:**
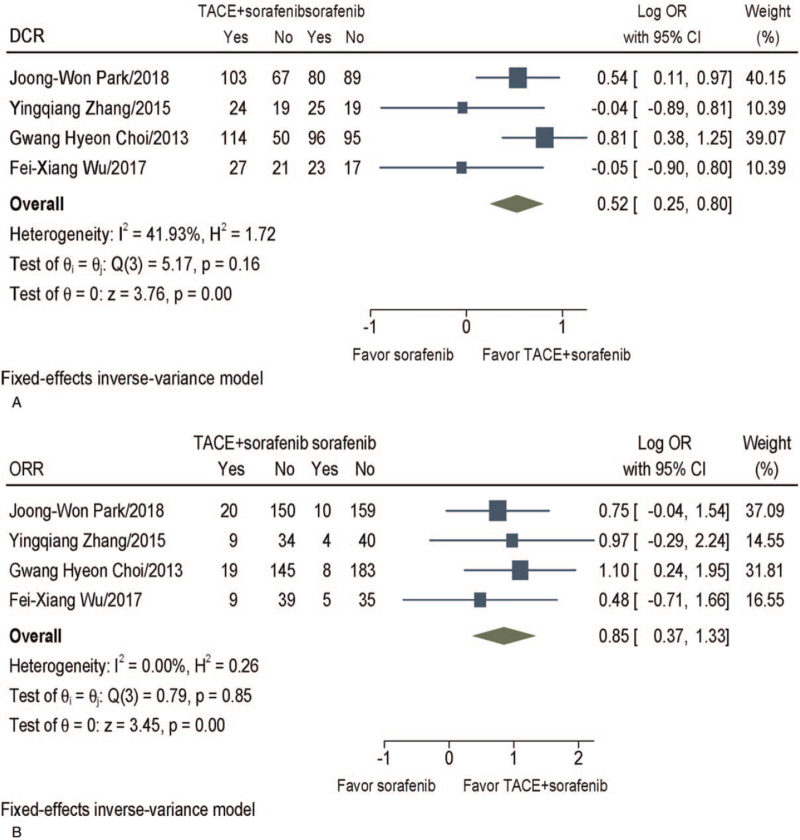
Meta-analysis of tumor response. (A) DCR. (B) ORR. CI = confidence interval, DCR = disease control rate, OR = odds ratio, ORR = objective response rate, TACE = transarterial chemoembolization.

Four studies^[16–19]^ reported radiographic tumor response, but only 3 of these studies used the modified guidelines for the response evaluation criteria in solid tumours criteria. The results showed the TACE + sorafenib group had a higher DCR (log OR, 0.52; 95% CI 0.25–0.80, *P* = .0002), ORR (log OR, 0.85; 95% CI 0.37–1.33, *P* = .0006) than sorafenib group. As no significant heterogeneity was observed (*I*^2^ = 41.93%, *P* = .16; *I*^2^ = 0%, *P* = .85, respectively), fixed-effect models were adopted to estimate the pooled log OR (Fig. 4).

### 3.5 Safety and toxicity (grade 3 or 4)

#### 3.5.1 Hand–foot skin reaction

**Figure 5:**
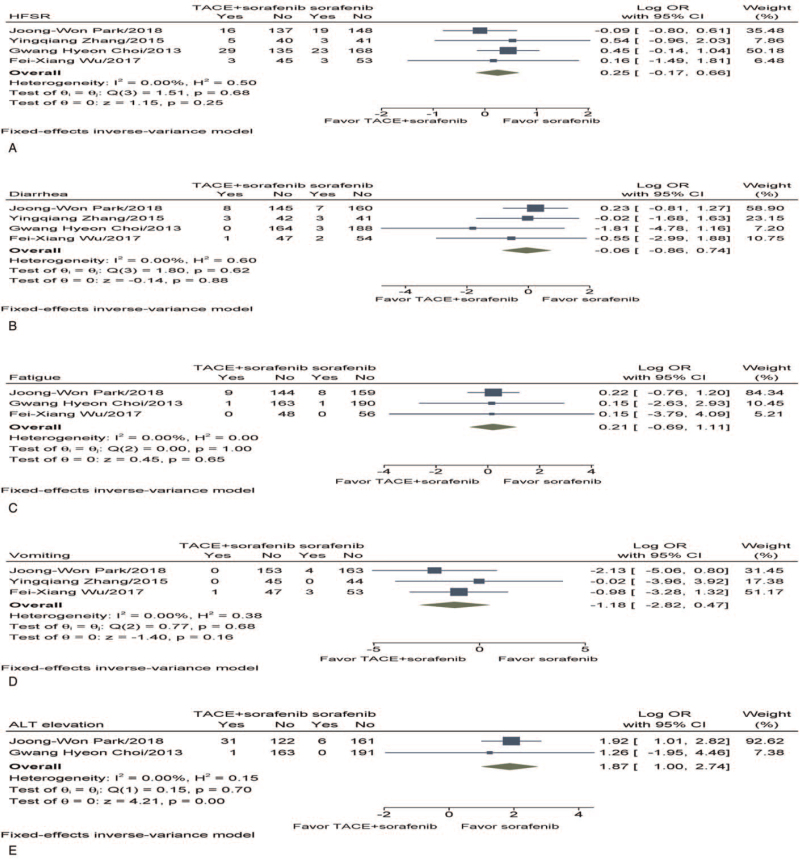
Meta-analysis of safety and toxicity (Grade 3 or 4). (A) HFSR. (B) Diarrhea. (C) Fatigue. (D) Vomiting. (E) ALT elevation. ALT = alanine aminotransferase, CI = confidence interval, HFSR = hand–foot skin reaction, OR = odds ratio, TACE = transarterial chemoembolization.

Four studies^[16–19]^ reported hand–foot skin reaction (HFSR). As significant homogeneity was observed (*I*^2^ = 0%, *P* = .68), a fixed-effect model was employed to estimate the combined log OR. Pooled data demonstrated that there was no observable difference found in HFSR between the groups (log OR, 0.25; 95% CI −0.17–0.66, *P* = .25) (Fig. 5A).

#### 3.5.2 Diarrhea

Four studies^[16–19]^ reported diarrhea. As significant homogeneity was observed (*I*^2^ = 0%, *P* = .62), a fixed-effect model was applied to estimate the pooled log OR. There was no observable difference found in diarrhea between the 2 groups (log OR, −0.06; 95% CI −0.86–0.74, *P* = .88) (Fig. 5B).

#### 3.5.3 Fatigue

Three studies^[16,18,19]^ reported fatigue. As no significant heterogeneity was observed (*I*^2^ = 0%, *P* = 1.00), a fixed-effect model was applied to estimate the pooled log OR. Pooled data revealed that there was no observable difference found in fatigue between the groups (log OR, 0.21; 95% CI −0.69–1.11, *P* = .65) (Fig. 5C).

#### 3.5.4 Vomiting

Three studies^[17–19]^ reported vomiting. As significant homogeneity was observed (*I*^2^ = 0%, *P* = .68), a fixed-effect model was employed to estimate the combined log OR. There was no observable difference found in vomiting between the groups (log OR, −1.18; 95% CI −2.82–0.47, *P* = .16) (Fig. 5D).

#### 3.5.5 Alanine aminotransferase elevation

Two studies^[16,19]^ reported alanine aminotransferase (ALT) elevation. As no significant heterogeneity was observed (*I*^2^ = 0%, *P* = .70), a fixed-effect model was adopted to estimate the pooled log OR. Pooled data indicated that TACE + sorafenib had significantly higher elevated ALT (log OR, 1.87; 95% CI 1.00–2.74, *P* < .001) than those treated with sorafenib (Fig. 5E).

## 4 Discussion

As we all know, the incidence of liver cancer is increasing, and it is estimated that 1 million people at least will die from the disease every year by 2030.^[28]^ HCC, as the leading type of PLC, has a poor prognosis with a 5-year OS rate is less than 20%, and the patients’ survival depends on the stage of the disease.^[29]^ Although TACE and sorafenib are recommended by international researchers and scholars as first-line treatment strategies for intermediate-stage HCC and advanced HCC, respectively, and they have been proven to be safe and effective,^[3,9,15,30]^ for patients with intermediate–advanced stages, there is controversy regarding the clinical evaluation results between the combined application of these 2 nonradical methods and the single use of sorafenib. This meta-analysis aimed to compare the effectiveness and safety between TACE + sorafenib and sorafenib in the management of intermediate–advanced HCC.

We also found that TACE + sorafenib has significantly longer OS and TTP than sorafenib, which is consistent with the viewpoint of Choi et al,^[16]^ who concluded based on their propensity score analysis of 192 patients that the TACE + sorafenib group was associated with a significantly longer OS and TTP than sorafenib and that of Kok et al^[20]^ Several possible mechanisms may explain the complementary effects of TACE plus sorafenib.

Firstly, we found that the treatment time of sorafenib in the combined group was longer than that of patients receiving sorafenib monotherapy, which may be the reason for the better response rate and longer survival time of the former.^[16]^ TACE embolization achieves the purpose of treatment by reducing the blood supply to the cancer foci and continuously releasing chemotherapeutic drugs. However, the side effects of TACE include overexpression of VEGF (because TACE can induce ischemic or hypoxic changes),^[31]^ impaired liver function, and recurrence of liver cancer. Comfortingly, sorafenib blocks several core processes in tumor growth and progression. To be specific, it can inhibit the tyrosine kinase of the VEGF signaling pathway, thereby reducing tumor angiogenesis and RAF kinase, resulting in a decrease in cell proliferation.^[31]^ Therefore, the combination of TACE plus sorafenib reduces angiogenesis.^[32,33]^ Secondly, TACE and sorafenib may affect the prognosis of HCC through various complex abnormal regulation of miRNA.^[34]^ Thirdly, sorafenib may ameliorate lipiodol retention in HCC,^[35]^ which offers support for better local control of advanced HCC. In summary, TACE combined with sorafenib can significantly improve the survival benefits of patients with intermediate–advanced HCC through the above mechanisms.

Four studies^[16–19]^ reported radiographic tumor response. We found that TACE + sorafenib group had a higher DCR (log OR, 0.52; 95% CI 0.25–0.80, *P* = .0002), ORR (log OR, 0.85; 95% CI 0.37–1.33, *P* = .0006) than sorafenib group. This is consistent with previous studies reporting patients treated with TACE + sorafenib to have a DCR of up to 80.5% and an ORR of up to 57%,^[19,33,36]^ and patients treated with sorafenib have a DCR of up to 43% and an ORR of up to 3.3%.^[31,37]^

Regarding adverse events, we found no significant difference in terms of HFSR, diarrhea, fatigue, vomiting between the 2 groups. Both therapies can provide patients with complications relief, confirming the value of these therapies in improving the quality of life. For grade 1 or 2 adverse events, we did not make a comparison between the 2 groups, because these symptoms can be significantly alleviated by corresponding symptomatic treatment. Herein, we mainly extracted grade 3 or 4 complications for comparison. Tolerability to simultaneous TACE and sorafenib therapies is good, as adverse events or complications between the sorafenib monotherapy and the combination group were comparable. Unfortunately, compared with sorafenib, TACE + sorafenib was associated with increased occurrence of elevated ALT (log OR, 1.87; 95% CI 1.00–2.74, *P* < .05). This may be closely related to the damage of liver function by TACE. Embolism syndrome is the most common after TACE, mainly manifested as fever, liver pain, nausea, and vomiting. There may also be transient liver dysfunction, white blood cell decline, kidney damage, bleeding at the puncture site, and dysuria. Adverse reactions after interventional embolization usually last for a short time, about 5 to 7 days.^[38,39]^ In summary, the incidence of complications in both treatment groups was low, which is consistent with some previous studies^[16,18]^ indicating the safety of TACE + sorafenib and sorafenib for intermediate–advanced HCC.

The above findings suggest that the combined group of TACE + sorafenib may be superior to sorafenib in the medical treatment of unresectable HCC. Compared with sorafenib, TACE + sorafenib appears to offer improved OS and tumor response, with comparable adverse effects. Future studies also should consider medical expenses, and quality of life and consider whether certain subgroups benefit from different combinations between TACE and sorafenib.

We should face these findings with caution because of some limitations of this study. First, most of the included literature (5 of 6, 83%) forms the basis of this meta-analysis, and these retrospective cohort designs have a relatively high level of inherent bias (eg, selection bias, measurement bias, and observation bias). Second, there are differences in the mean daily dose of sorafenib and the mean sorafenib administration time between the 2 groups. One study^[19]^ gave these records in detail, while other studies did not. Also, there are differences in the infusion chemotherapy drugs (doxorubicin or cisplatin or epirubicin) and embolic materials (eg, gelatin sponges or gel foam) used in different studies, and these factors may affect the pooled results. Thirdly, although these studies set eligibility criteria to include research subjects, they cannot fully guarantee that the baseline characteristics between groups are as balanced as possible like RCT. Without those specific details, it is difficult for us to evaluate and control the source of bias. Therefore, these above limitations highlight the need for larger RCTs to affirm this finding and clarify whether certain subgroups benefit from different combinations between TACE and sorafenib.

## 5 Conclusions

Although the TACE + sorafenib group has a higher elevated ALT, our meta-analysis revealed survival benefits associated with treating intermediate–advanced HCC with TACE + sorafenib instead of sorafenib monotherapy. Besides, the TACE + sorafenib group provided a higher hepatic tumor response rate compared to the sorafenib group. Future studies are warranted to confirm this finding and clarify whether certain subpopulations benefit from different a combination between TACE and sorafenib.

## Author contributions

**Conceptualization:** Yong Xie, Huan Tian.

**Data curation:** Yong Xie, Huan Tian, Jian Liu, Zhuoyan Cai.

**Formal analysis:** Yong Xie, Huan Tian, Jian Liu.

**Methodology:** Yong Xie, Huan Tian, Bin Xiang.

**Software:** Yong Xie, Huan Tian, Jian Liu.

**Supervision:** Bin Xiang, Yongjin Zhang, Hua Xiang.

**Writing – original draft:** Yong Xie, Huan Tian.

**Writing – review & editing:** Bin Xiang, Yongjin Zhang, Hua Xiang.
